# Two-hit events occurred independently in bilateral breast cancers in a germline double heterozygous carrier for* BRCA1* and *BRCA2*

**DOI:** 10.1007/s12282-025-01740-4

**Published:** 2025-07-02

**Authors:** Ryoko Semba, Hidetaka Eguchi, Mizuki Takatsu, Toko Hashizume, Hideaki Moteki, Kazuma Maeno, Fumi Murakami, Junichiro Watanabe, Goro Kutomi, Masami Arai

**Affiliations:** 1 Department of Breast Surgery, Juntendo University Nerima Hospital, Takanodai 3-1-10, Nerima-Ku, Tokyo 177-8521 Japan; 2 Diagnostics and Therapeutics of Intractable Diseases, Intractable Disease Research Center, Juntendo University Graduate School of Medicine, Tokyo, Japan; 3 Department of Clinical Genetics, Aizawa Hospital, Matsumoto, Japan; 4 Department of Breast and Thyroid Surgery, Aizawa Hospital, Matsumoto, Japan; 5 Department of Breast and Endocrine Surgery, Suwa Red Cross Hospital, Suwa, Japan; 6 Department of Breast Oncology, Juntendo University Graduate School of Medicine, Tokyo, Japan; 7 Department of Clinical Genetics, Juntendo University Graduate School of Medicine, Hongo 2-1-1, Bunkyo-Ku, Tokyo 113-8421 Japan

**Keywords:** Hereditary breast and ovarian cancer, Breast cancer, *BRCA1* and *BRCA2*, Double heterozygosity, Two-hit events

## Abstract

While patients with hereditary breast and ovarian cancer with germline double heterozygosity (GDH) for *BRCA1* and *BRCA2* are rare, carcinogenesis in these cases remains unclear. We examined two-hit events of heterochronous bilateral breast cancers in a patient with GDH for *BRCA1* and *BRCA2*. A 65-year-old woman developed right breast cancer (triple-negative type) at the age of 49 and left breast cancer (triple-negative type) at 55. Family history indicated that multiple relatives on her mother’s side also developed breast cancer. *BRCA1/2* genetic testing (BRACAnalysis®) showed that she had variants in both the *BRCA1* and *BRCA2* (*BRCA1*:c.5193 + 2dup, *BRCA2*:c.6952C > T/p.Arg2318Ter). According to the data from the test, the former was interpreted as likely pathogenic at Myriad Inc. Further examination regarding two-hit events in her bilateral breast cancers was obtained by somatic mutation analysis using DNA isolated from cut slide specimens of formalin-fixed and paraffin-embedded tumor samples. We first confirmed the pathogenicity of the *BRCA2* variant by detecting unusual splicing of *BRCA2* that entirely skipped exon 19 using cultured T cells of the proband. Loss of heterozygosity in *BRCA1* was observed in her right breast cancer. On the other hand, a somatic nonsense pathogenic variant in *BRCA2* (variant allele frequency = 15%) and a two-hit event in *APC* (VAF = 80%) were also found in her left breast cancer. These data provide evidence of different carcinogenesis between left and right breast cancer. Clinical and pathogenic characteristics of cancers with GDH for *BRCA1* and *BRCA2* depend on the genes somatically mutated in wild alleles.

**Supplementary Information:**

The online version contains supplementary material available at 10.1007/s12282-025-01740-4.

## Introduction

Hereditary breast and ovarian cancer (HBOC) syndrome is an autosomal dominant cancer susceptibility disorder caused by deleterious germline variants in *BRCA1* or *BRCA2* [1]. The cumulative risk of breast cancer at 70 years is 64.6% for *BRCA1* germline pathogenic variants (PVs) carriers and 61.0% for *BRCA2* PVs carriers [2]. In Japan, the management of PVs carriers with *BRCA1* or *BRCA2* with risk-reducing surgeries and surveillance with magnetic resonance imaging has been covered under public medical insurance since 2020. The number of genetic tests ordered doubled compared with the previous year in Japan [3].

Based on the 2020 Japanese nationwide registration data, *BRCA1* or *BRCA2* PVs were found in 719 (10.2%) or 638 (9.1%) of the 7043 registered cases that met the screening criteria for genetic testing for HBOC. Additionally, seven cases (0.5%) with germline double heterozygosity (GDH) for *BRCA1* and *BRCA2* were also registered [4].

To our knowledge, there are no previous reports on the two-hit status of tumor suppressor genes, including *BRCA1* and *BRCA2,* in breast cancers in patients with GDH for *BRCA1* and *BRCA2*. We examined the carcinogenesis of two primary breast cancers in the proband by analyzing somatic mutations in cancer-associated genes including *BRCA1* and *BRCA2*.

## Patients and methods

**Fig. 1:**
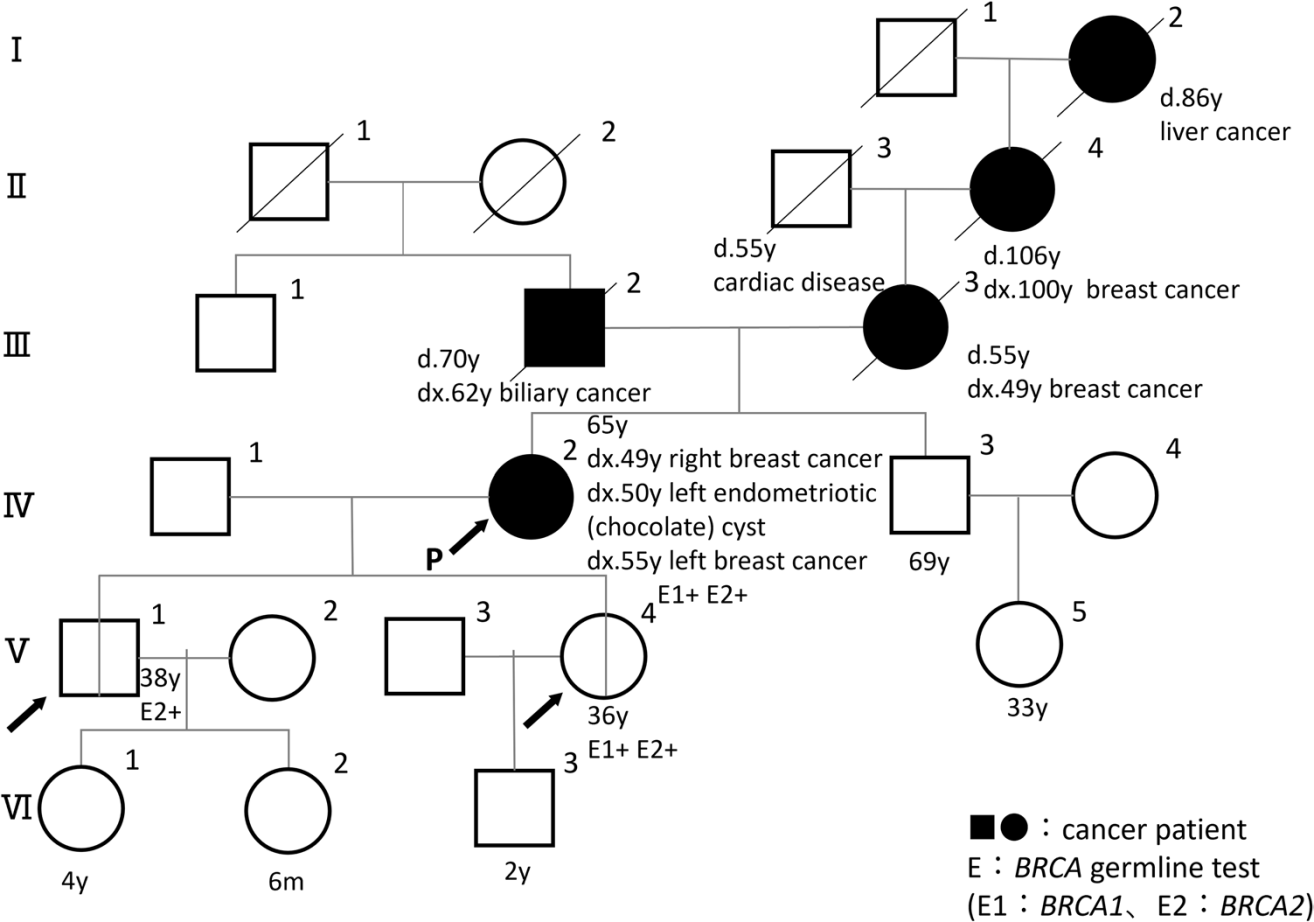
Pedigree of the proband. Filled circles and rectagles indicate cancer-prone patients

A 65-year-old woman was diagnosed with right breast cancer (papillary ductal carcinoma, triple negative type) at age 49 and left breast cancer (medullary carcinoma, triple negative type) at 55. Histopathological results of right and left breast cancers were summarized in Supplementary Table 1. The cystic tumor of the left ovary was confirmed to be an endometriotic (chocolate) cyst based on the histopathological findings from the laparoscopic left salpingo-oophorectomy. Her family history is shown in Fig. 1. BRACAnalysis® revealed that she had two potential PVs in both the *BRCA1* and *BRCA2* genes. The *BRCA2* variant, NM_000059.4(*BRCA2*):c.6952C > T(p.Arg2318Ter) was a nonsense variant with a definite pathogenicity registered at the ClinVar database reviewed by an expert panel (status of three stars). The *BRCA1* variant, NM_007294.4(*BRCA1*):c.5193 + 2dup, was a variant on the splice donor site with duplication of thymidine nucleotide at the canonical splicing donor site ‘gt’ in intron 19. This variant was deemed likely pathogenic based on Myriad’s internal data, though the evidence has not yet been published.

To confirm the pathogenicity of the germline *BRCA1* variant, we performed reverse transcription-polymerase chain reaction (RT-PCR) to evaluate the splicing abnormality.

Total RNA was prepared from the cultured T cells from the proband’s peripheral blood with or without treatment with 10 μg/mL of puromycin for 5 h using the NucleoSpin RNA plus kit (Macherey–Nagel GmbH, Düren, Germany). Total RNA was subjected to cDNA synthesis using the ReverTra Ace enzyme (Toyobo Inc., Osaka, Japan) following the manufacturer’s instructions. Primers, BRCA1_RT_Ex18_F01: ATG CTG AGT TTG TGT GTG AAC and BRCA1_RT_Ex21_R01: CCC ATA GCA ACA GAT TTC TAG C and AmpliTaq Gold™ 360 Master Mix (Thermo Fisher, Waltham, MA, USA) were used for the amplification. After electrophoresis on the 1.5% agarose gel, the bands were visualized using blue light illumination. Sanger sequencing was conducted with BigDyeTerminator 3.1 (Thermo Fisher) and SeqStudio sequencer (Thermo Fisher).

To further explore the second hit events, we conducted mutation analysis by panel sequencing of 61 selected cancer-related genes, including *BRCA1* and *BRCA2*, using the formalin-fixed and paraffin-embedded bilateral cancer tissue specimens of the patient. We collected cancerous portions using a laser microdissection system, Leica LMD 7000 (Leica Microsystems GmbH Wetzlar, Germany). DNA was extracted from the dissected tissue using a FormaPure XL DNA kit (Beckman Coulter Inc, South Kraemer Boulevard Brea, CA, USA). Before the library construction, the extracted DNA was treated with Uracil-DNA Glycosylase (UNG), Heat-labile (Toyobo) according to the manufacturer’s recommendation to reduce sequence artifacts. Panel sequencing was conducted as described previously [5]. Briefly, AmpliSeq Custom panel was designed using AmpliSeq 7.0.2 (Thermo Fisher Scientific) that covers exons, exon–intron boundaries, 5’UTR and 3’UTR regions of 61 genes associated with hereditary cancer including *BRCA1* and *BRCA2.* Ion GeneStudio S5 Plus sequencer (Thermo Fisher Scientific) was used to obtain the sequence data. The obtained data were analyzed using the Ion Reporter server 5.10 (Thermo Fisher Scientific). The called variants were reviewed by visual inspection on the Integrative Genomics Viewer (Broad Institute).

## Results

**Fig. 2:**
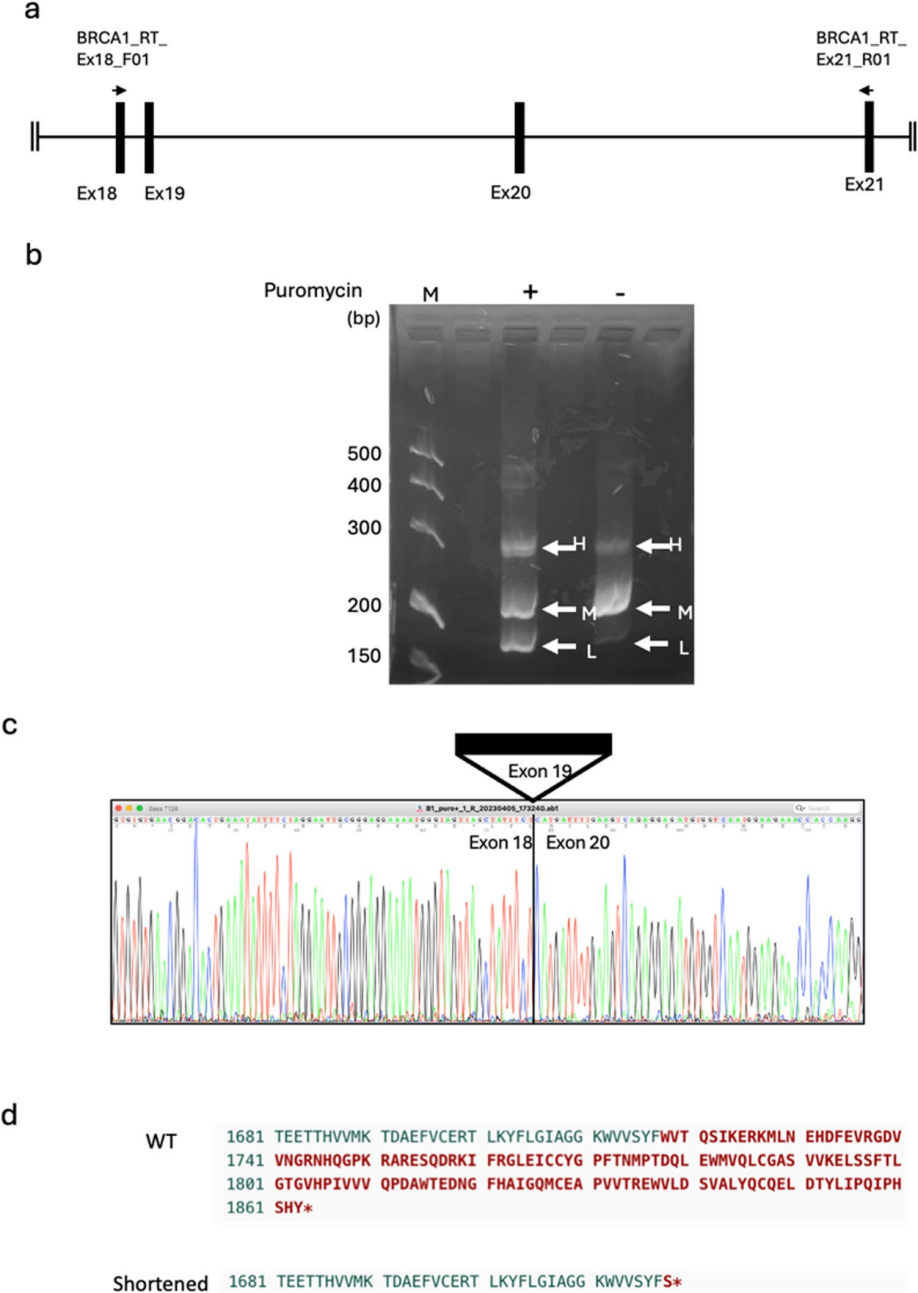
Reverse-transcription-polymerase chain reaction (RT-PCR) analysis of *BRCA1* using RNA from the proband’s cultured T cells **a** Schematic diagrams of locations of primers used for the RT-PCR analysis. Vertical line indicates exons of *BRCA1*. **b** Agarose gel electrophoresis of amplified products of RT-PCR. To prevent nonsense-mediated mRNA decay (NMD), the T cells were treated with puromycin for 5 h before cell collection, and aberrant transcripts migrating faster (L) and slower (H) clearly appeared. M: Wild type, L: Shortened products generated by aberrant splicing to skip entire exon 19, H: heteroduplex formed by wild type and the shortened products. DNA Marker 500/S (Rikaken Co., Tokyo, Japan) was run as a DNA size marker (M). **c** Electrograms of the Sanger sequencing of the RT-PCR products L. Skipping of the entire exon 19 was observed. **d** Amino acid sequences of the wild type (WT) and shortened BRCA1 protein. One-letter amino acids marked with red color in the WT indicate 146 amino acids missing in the shortened BRCA1 protein generated in the proband

To confirm the internal data from Myriad Inc., we first conducted RT-PCR analysis of *BRCA1* using RNA from cultured T cells of the proband. Figure 2a shows the location of primers and exons of *BRCA1*. We found faster (L) and slower (H) migrated products in the agarose gel electrophoresis in addition to the wild type (M); these aberrant products were evident when the cells were treated with puromycin, suggesting that the aberrant products were efficiently destroyed by a nonsense-mediated decay system (Fig. 2b). On the other hand, products L and H were faintly obserbed in the cells without treatment of puromycin. These observations suggested that the aberrant products were efficiently destroyed by a nonsense-mediated decay system (Fig. 2b). Along with this hypothesis, when analyzing RNA prepared from the peripheral blood cells directly, we did not observe such aberrant transcripts in the proband (data not shown). Sanger sequencing demonstrated that the L products correspond to the abnormal *BRCA1* transcripts skipping entire exon 19 (Fig. 2c), while the H products were heteroduplex of the wild type and shorter products lacking exon 19 as was evident by Sanger sequencing (data not shown). Skipping exon19 resulted in lacking 146 amino acids coded by the exons 19 and 20 and the generation of a premature stop codon, p.Trp1718Serfs*2 due to a frameshift of *BRCA1* (PVS1, PS3,) (Fig. 2d). The C-terminus of the BRCA1 BRCT domain is known to be essential for its function [6]; thus, this truncation is expected to be deleterious. This variant is not registered in a Japanese genome database for healthy individuals, ToMMo jMorp 60KJPN, nor in the international gnomAD v4.1.0 (PM2). Based on these results, the pathogenicity of this variant was reclassified as “pathogenic” based on the ACMG/AMP guidelines (PVS1, PS3, PM2, PM4,) [7]. *BRCA2*:c.6952C > T/p.Arg2318* has been most frequently identified in Japaneser individuals heterozygous for HBOC based on JOHBOC database, as well as in one person with Fanconi anemia [4, 8–10]. Taken together, the proband had confirmed pathogenic variants in both *BRCA1* and *BRCA2*. Carrier diagnosis was then performed for her unaffected son and daughter. The results of two kinds of single-site testing carried out at a registered clinical laboratory showed that her son had only the *BRCA2* gene variant (V-1 in Fig. 1), while her daughter possessed both the *BRCA1* and *BRCA2* gene variants (V-4 in Fig. 1).

We considered that it is clinically valuable to ascertain whether the same molecular carcinogenesis is present in bilateral breast cancer. Thus, we conducted somatic mutation analysis of each breast cancer of the proband to evaluate the involvement of these germline variants in carcinogenesis.

**Fig. 3:**
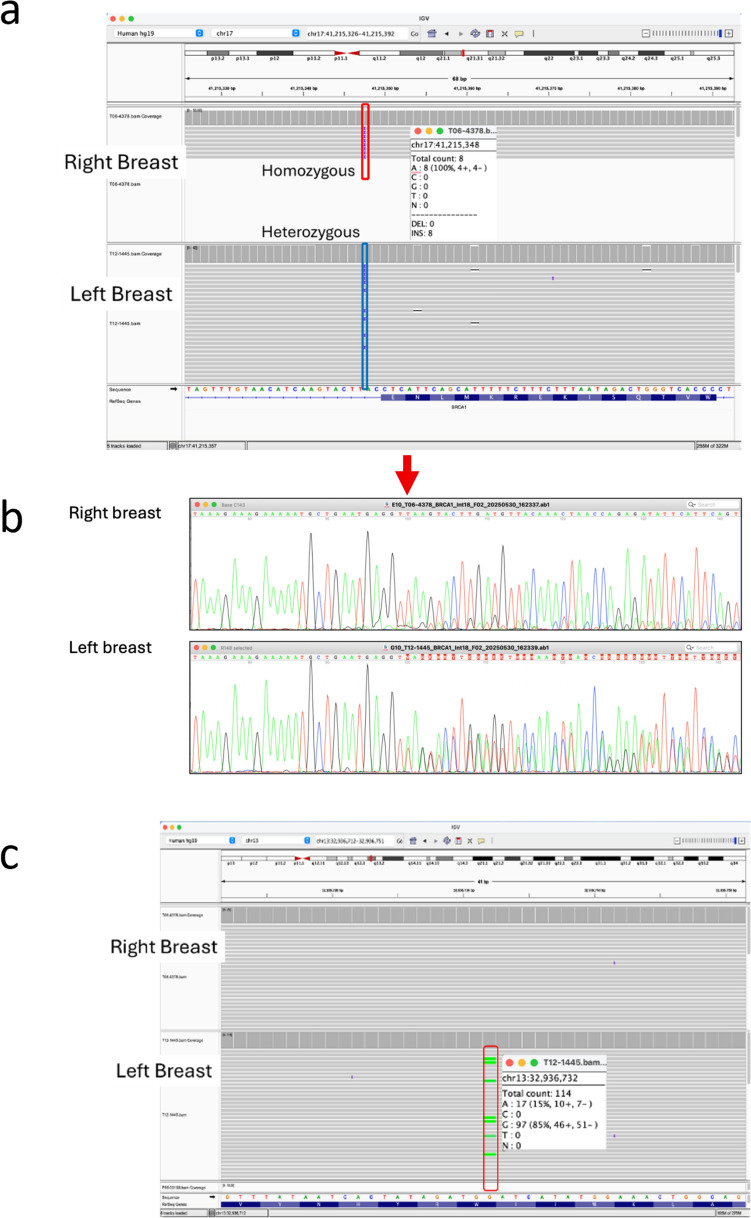
Results of panel sequencing of *BRCA1* and *BRCA2* germline variants in the right and left breast cancer of the proband. **a** Loss of heterozygosity (LOH) at the germline splice site variant, *BRCA1*:c.5193 + 2dup, was observed in the right breast cancer as shown by Integrative Genomics Viewer (IGV). Sanger sequencing of the regions surrounding the germline splice site variant, *BRCA1*:c.5193 + 2dup in the right and left breast cancer of the proband. The allele harboring the splice site variant, *BRCA1*:c.5193 + 2dup was much dominant in right breast cancer as compared to the left breast one. The germline nonsense variant, *BRCA2*:c.6952C > T/p.Arg2318* was completely lost, while a wild-type allele remained intact in the right breast cancer as shown by IGV

In the right breast cancer, loss-of-heterozygosity (LOH) was clearly observed at the splice site variant *BRCA1*:c.5193 + 2dup, indicating loss of the wild-type allele. This was confirmed by the Sanger sequencing (Fig. 3b). In the left breast cancer, the duplicated variant, *BRCA1*:c.5193 + 2dup along with the wild type sequences were clearly shown as heterozygous, while only faint peaks of the wild type sequence derived from the contaminated normal tissue was detected in the right breast cancer (Fig. 3b). However, the germline nonsense variant *BRCA2*:c.6952C > T/p.Arg2318Ter was completely lost, while a wild-type allele remained intact in the right breast cancer (Fig. 3c). Therefore, second-hit events occurred in *BRCA1* but not in *BRCA2* in the right breast cancer of the proband.

**Fig. 4:**
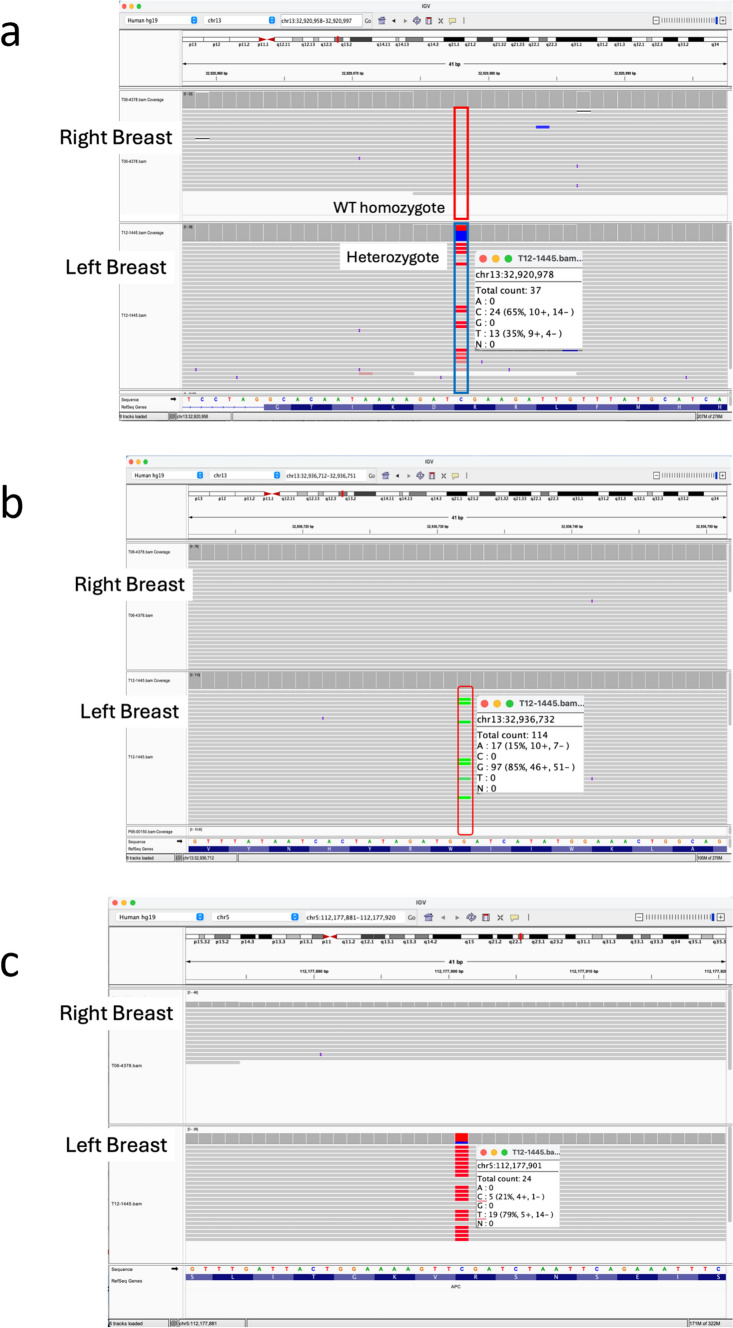
Somatic mutation analysis results for the right and left breast cancer of the proband. **a**. The germline variant, *BRCA2*:c.6952C > T/p.Arg2318* was completely lost in the right breast cancer. **b** Next-generation sequencing (NGS) analysis identified a nonsense somatic mutation in *BRCA2*. A somatic nonsense mutation, *BRCA2*:c.7878G > A/p.Trp2626* was observed in the left breast cancer. **c** Sequence analysis result of the *APC* by NGS. A somatic nonsense mutation, *APC*:c.6610C > T/p.Arg2204* was observed in the left breast cancer. This variant showed approximately 80% of variant allele frequency, indicating that the wild-type allele of *APC* gene was mainly deleted

In the left breast cancer, we found a somatic nonsense mutation *BRCA2*:c.7878G > A/p.Trp2626* with variant allele frequency (VAF) of 15% in addition to the germline nonsense variant, c.6952C > T/p.Arg2318Ter. In the right breast cancer, the *BRCA2* allele horboring the germline variant was completely lost, and no additional somatic mutations with pathogenic/likely pathogenic were identified in *BRCA2* (Fig. 4a, b). In contrast, no somatic deleterious mutations were detected in *BRCA1* in the left cancer (data not shown). In addition to the genetic event in the *BRCA2*, a somatic nonsense mutation, *APC*:c.6610C > T/p.Arg2204* was also detected with a high VAF of 79% (Fig. 4c). Then, second-hit events occurred in *BRCA2* and *APC* in the left breast cancer.

## Discussion

To our knowledge, this paper is the first to provide evidence of differing carcinogenic processes in a patient with GDH for *BRCA1* and *BRCA2* between right and left breast cancer by somatic mutation analysis, expanding the target to the main tumor suppressor genes. Most importantly, breast carcinogenesis in HBOC with GDH for *BRCA1* and *BRCA2* depends on each mutation event in tumor suppressor genes, including *BRCA1* or *BRCA2*, but not on common mechanisms in GDH carriers with both *BRCA1* and *BRCA2*.

Seven cases with GDH for *BRCA1* and *BRCA2* were registered in JOHBOC (2020), corresponding to 0.5% of *BRCA1* or *BRCA2* pathogenic variant (PV) carriers (7/1364) [4]. The dataset of CIMBA, the International Research Consortium for HBOC, included 93 individuals (0.29%) from 84 families of 32,295 female carriers with *BRCA1/2* PVs; of cases with GDH for *BRCA1* and *BRCA2*, 45.2% were involved in three common germline PVs in Ashkenazi [11]. The frequency of *BRCA1* and *BRCA2* GDH carriers in our country is not as rare as that of foreign countries in CIMBA.

The germline *BRCA1* variant, c.5193 + 2dup in the proband was classified as “likely pathogenic” in the BRACAnalysis® test report of the proband by Myriad. Recently, Ambry Genetics registered this variant in the ClinVar database as “pathogenic” based on their internal data that this variant results in abnormal splicing in the set of samples tested [12]. We confirmed their internal data by conducting RT-PCR analysis using total RNA prepared from cultured T cells of the proband treated with puromycin. When conducting an in silico prediction tool for splicing, the SpliceAI tool [13] gave a delta score of 0.96 in donor loss, strongly suggesting splicing alteration. SPiCE v2.1 [14] also predicted the loss of splicing donor, while Human Splicing Finder Pro (Genomnis, Marseille, France) demonstrated no significant impact on splicing signals. Thus, confirming the splicing alteration in the proband’s material was very important. We provide clear evidence that this variant induced the skipping of the entire exon 19 to generate aberrant RNA that was destroyed via a nonsense-mediated decay system in the peripheral blood cells. This finding supports the submission of Ambry Genetics at ClinVar based on their internal data.

Patients with GDH for *BRCA1* and *BRCA2* were first reported in a Hungarian HBOC family [15]. Clinical and pathological studies of breast cancers in Korean patients with GDH for *BRCA1* and *BRCA2* revealed that the age of onset was younger than that of patients with a single germline PV of *BRCA1* or *BRCA2* and the probands had a more dense family history of breast cancer [16]. Additionally, pathological findings in terms of estrogen and progesterone receptor status and dominance of triple-negative breast cancer among subtypes were similar to those with *BRCA1* single PV [16].

In the family of the proband, there are three patients with breast cancer: herself, her mother, and her maternal grandmother. Their onset age of breast cancer was 49, 49, and 100 years, respectively. The proband’s onset age was not less than the average age of a single germline pathological variant carrier (BRCA1: 40.2 years, BRCA2: 41.7 years) of Japanese patients with breast cancer with either *BRCA1* or *BRCA2* [1]*.* In contrast, the pathological findings of both bilateral breast cancers of the proband were triple negative, a typical characteristic of breast cancer with *BRCA1* germline PV carriers. This was consistent with the findings of a previous study [16].

In this case, somatic mutation analysis in breast cancer tissue specimens demonstrated that the pathogenesis may differ between right and left breast cancer. Right breast cancer is associated with *BRCA1* inactivation. In contrast, *BRCA2* and *APC*, typical tumor suppressor genes, may be involved in left breast cancer carcinogenesis. A study examined the LOH of *BRCA1* and *BRCA2* in 12 breast and ovarian cancer specimens with GDH and concluded that there was no specific pattern in the two-hits status of *BRCA1* and *BRCA2* [11]. They reported that three cases (one *BRCA1* and two *BRCA2* cases) of breast cancer and one case in *BRCA1* of ovarian cancer showed LOH, but they could not find any LOH in the remaining cases. In a Japanese breast cancer case with GDH for *BRCA1* and *BRCA2*, immunohistochemical analysis of breast cancer tissue of the patient revealed loss of both BRCA1 and BRCA2 proteins; therefore, both alleles of *BRCA1* and *BRCA2* might simultaneously be inactivated [17]. In contrast, there were no cases of both inactivation of *BRCA1* and *BRCA2* in Rebbeck’s report [11]. In our case, each tumor had its own carcinogenesis process. Specifically, in the left breast cancer, *APC* inactivation was observed in addition to the *BRCA2* inactivation. *APC* somatic mutation has been found in 18% of breast cancer cases; the mutations were significantly more frequent in higher, advanced stages, suggesting that *APC* mutations are associated with tumor progression in breast cancers [18]. Since some cases cannot be explained solely by two-hit events in the *BRCA1* and *BRCA2* genes [11], we extended the extent of target genes for two-hit to other main tumor suppressor genes. This enabled us to comprehensively show the left breast cancer carcinogenesis.

From our findings in this study, it revealed that two-hit event of tumor suppressor gene is not necessarily restricted to generate in an allele of causasive gene with a pathogenic variant. Germline genetic testing such as BRACAnalysis®　may not reflect inactive status of the tumor suppressor gene in his or her tumor. Tumor acquired heterogeneity as it grows. It could be cause the discrepancy between the result of companion diagnosis and the effect of molecular targeted drugs.

As multi-gene panel testing becomes more widespread, the possibility that multiple pathogenic variants are detected will be enlarged in clinical practice. These variants are transmitted based on Mendelian inheritance. We should take care of the possibility that there remaind the existence of another pathological variant, if the clinical phenotype cannot be completely explained by the result of genetic testing, however, it is difficult in similar functional genes such as *BRCA1* and *BRCA2*.

In conclusion, we present evidence of independent carcinogenesis of bilateral breast cancer developed in a Japanese patient with HBOC who harbored GDH for *BRCA1* and *BRCA2*.

## Supplementary Information

Below is the link to the electronic supplementary material.

Supplementary file1 Supplementary Table. 1 Detailed histopathological results of left and right breast cancers (TIF 164 KB)

## Data Availability

The datasets generated and analyzed during the current study are not publicly available due to patient privacy considerations, but are available from the corresponding author on reasonable request.
