# Supplementary figures and images for: Two-hit events occurred independently in bilateral breast cancers in a germline double heterozygous carrier for BRCA1 and BRCA2
Source: Breast Cancer. 2025 Jul 2;32(6):1472–9. doi: 10.1007/s12282-025-01740-4 (PMC12552342; doi:10.1007/s12282-025-01740-4)

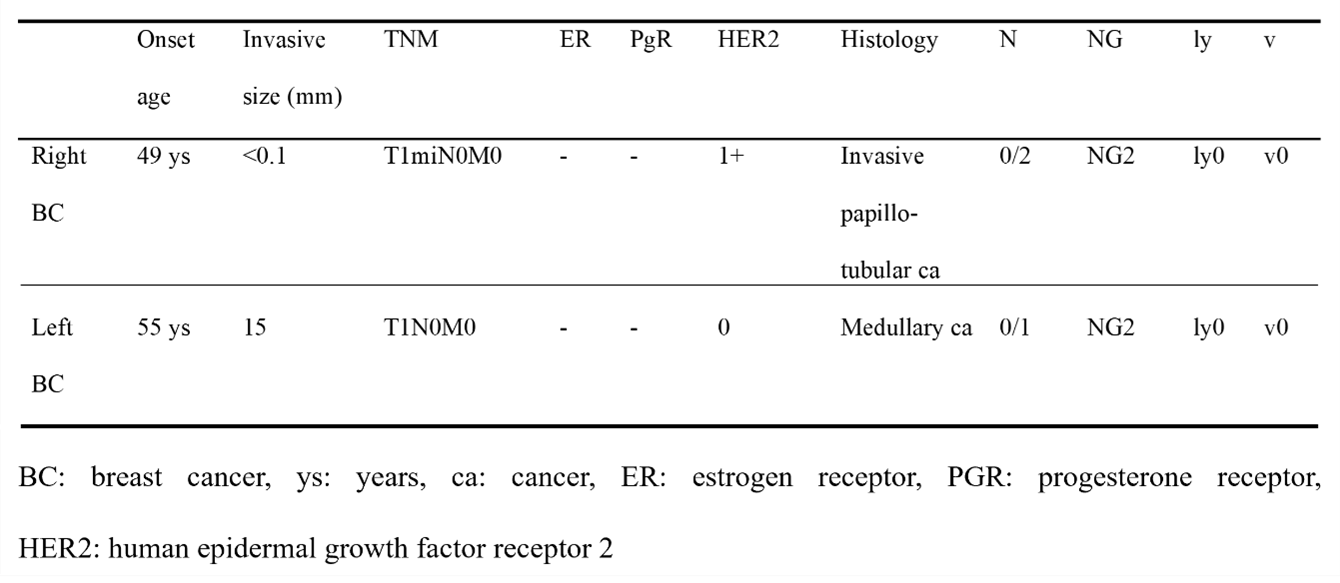

Supplement: Supplementary file 1 — Supplementary file1 Supplementary Table. 1 Detailed histopathological results of left and right breast cancers (TIF 164 KB) [file 12282_2025_1740_MOESM1_ESM.tif]
